# Early Signs of Atherogenic Features in the HDL Lipidomes of Normolipidemic Patients Newly Diagnosed with Type 2 Diabetes

**DOI:** 10.3390/ijms21228835

**Published:** 2020-11-22

**Authors:** Christina E. Kostara, Eleuterio Ferrannini, Eleni T. Bairaktari, Athanasios Papathanasiou, Moses Elisaf, Vasilis Tsimihodimos

**Affiliations:** 1 Laboratory of Clinical Chemistry, Faculty of Medicine, University of Ioannina, 45110 Ioannina, Greece; chkostara@gmail.com (C.E.K.); ebairakt@uoi.gr (E.T.B.); 2 Institute of Clinical Physiology, National Research Council, 56124 Pisa, Italy; ferrannini@ifc.cnr.it; 3 Department of Internal Medicine, Faculty of Medicine, University of Ioannina, 45110 Ioannina, Greece; thanasis.papathanasiou@gmail.com (A.P.); melisaf54@gmail.com (M.E.)

**Keywords:** HDL, type 2 diabetes, coronary heart disease, lipidomics

## Abstract

Cardiovascular disease (CVD) is the major cause of death in patients with type-2 diabetes mellitus (T2DM), although the factors that accelerate atherosclerosis in these patients are poorly understood. The identification of the altered quantity and quality of lipoproteins, closely related to atherogenesis, is limited in routine to a pattern of high triglycerides and low HDL-cholesterol (HDL-C) and in research as dysfunctional HDLs. We used the emerging NMR-based lipidomic technology to investigate compositional features of the HDLs of healthy individuals with normal coronary arteries, drug-naïve; recently diagnosed T2DM patients with normal coronary arteries; and patients with recent acute coronary syndrome. Patients with T2DM and normal serum lipid profiles even at diagnosis presented significant lipid alterations in HDL, characterized by higher triglycerides, lysophosphatidylcholine and saturated fatty acids; and lower cholesterol, phosphatidylcholine, phosphatidylethanolamine, sphingomyelin, plasmalogens and polyunsaturated fatty acids, an atherogenic pattern that may be involved in the pathogenesis of atherosclerosis. These changes are qualitatively similar to those found, more profoundly, in normolipidemic patients with established Coronary Heart Disease (CHD). We also conclude that NMR-based lipidomics offer a novel holistic exploratory approach for identifying and quantifying lipid species in biological matrixes in physiological processes and disease states or in disease biomarker discovery.

## 1. Introduction

Cardiovascular disease (CVD) is the most common complication and the major cause of death in patients with type-2 diabetes mellitus (T2DM). Although some studies support the notion that T2DM is an established CVD equivalent [1], other data have shown that T2DM patients may have increased CVD risk but not to the degree observed in secondary prevention populations [2]. The factors that accelerate atherosclerosis in individuals with impaired carbohydrate metabolism are poorly understood. Although the role of hyperglycemia in the pathogenesis of atherosclerosis remains debatable, the concentrations of classic CVD risk factors in patients with diabetes seem to have an additive effect on the risk of ischemic events [3]. In the United Kingdom Prospective Diabetes Study (UKPDS) the factors that had the greatest predictive power for future CVD in T2DM patients were not the indices of glycemic control, but instead, the levels of low-density lipoprotein (LDL)- and high-density lipoprotein (HDL)-cholesterol [4], as they are identified in everyday routine, not just in research with the presence of compositionally altered and dysfunctional HDL particles [5].

The well recognised main antiatherogenic role of HDLs is the participation in reverse cholesterol transport (RCT) from peripheral tissues to the liver. Moreover, HDL has multiple additional atheroprotective mechanisms that include amelioration of endothelial dysfunction and antioxidative, anti-inflammatory and antiapoptotic effects [6]. Low levels of HDL-cholesterol in plasma are strongly inversely related to cardiovascular disease CVD risk. Given that most therapies targeted at raising plasma HDL-C did not result in a lower rate of cardiovascular events, a large body of recent research effort has focused not on raising HDL-C, but on improving HDL function mainly via understanding the reverse cholesterol transport with the ultimate goal of discovering new and reliable biomarkers and targets for the prevention and treatment of CVD [7,8,9,10,11].

HDL is structurally and functionally complex and related measurements other than HDL-C and apolipoprotein AI, mainly the functionality and composition of the particles, have been considered as determinative factors for assessing CVD risk [8]. The research assays for measuring HDL functionality can provide evidence of the ability of HDL to perform specific cardioprotective functions, but they can be difficult to standardize for routine diagnostic testing. Alterations in the lipid compositions of HDLs mainly occurring in pathological conditions closely affect their structure and endow the particles with abnormal biological properties. Since the function of HDL is highly associated with its composition, it has been considered that it may be more feasible to develop future compositional assays based on the measurement of specific HDL-associated constituents with established functional importance [7].

Lipidomics, a branch of metabolomics, is a relatively new field in systems biology and represents a transition from an individual lipid study to the analysis of global lipid composition in a biological matrix in order to better understand their role in pathophysiological processes [12,13,14]. The two most popular analytical techniques used in lipidomics are nuclear magnetic resonance (NMR) and mass spectrometry (MS) which, combined with pattern recognition approaches, also known as “chemometrics,” offer an innovative analytical strategy to extract valuable information for biomarker discovery in the context of different disease phenotypes. NMR-based lipid analysis, despite its lower sensitivity compared to MS and typically limited by overlapping signals, is a non-destructive tool with high analytical reproducibility that does not require extensive steps for sample preparation and offers easy identification of molecular moieties and direct quantitative information [15,16]. High-field NMR spectroscopy is a powerful and reliable tool with which to assess the molecular compositions of biological samples. NMR facilities are widely used in clinical trials for metabolomics investigations in order to rapidly recognise unusual metabolic patterns in patients suffering from a range of diseases and as diagnostic tools to detect various biomarkers, especially those related to metabolic disorders. Proton NMR (^1^H-NMR) provides quantitative screening of major lipid classes and has increasingly been used in lipidomic assessments as a rapid, simple and non-invasive detection method [14,17].

In the present study, we applied a ^1^H-NMR-based lipidomic approach to analyze the compositional features of HDL particles of healthy individuals with normal coronary arteries; drug-naïve patients with a recent diagnosis of T2DM and normal coronary arteries; and patients with recent acute coronary syndrome (ACS).

## 2. Results

### 2.1. Study Population

Major characteristics of the three study groups (patients with T2DM without Coronary Heart Disease (CHD), patients with CHD without T2DM and controls) are presented in Table 1. The groups were matched for age and serum lipid parameters to minimize their confounding effects on the analysis. As expected, fasting glucose and HbA_1c_ levels were higher in T2DM patients compared to controls and CHD patients. In addition, the prevalence of metabolic syndrome was higher in the 2 patient groups compared to controls, while metabolic syndrome was more prevalent in T2DM patients compared to those with CHD. Total and LDL-cholesterol values in T2DM and CHD patients were well above the guideline-assigned targets for these patient groups, but these were baseline values before the diagnosis of these conditions and before the use of any lipid-lowering therapy.

### 2.2. HDL Lipidome Identification

**Figure 1:**
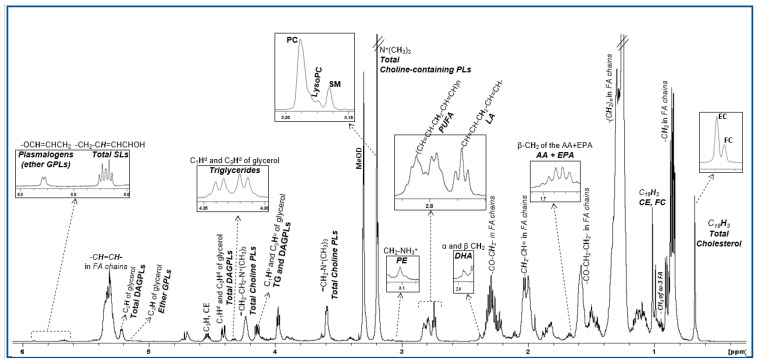
^1^H NMR spectrum of an HDL lipid extract. Peak assignments are summarized in Table S1.

The lipidomic profile of HDL produced by ^1^H NMR spectroscopy contains signals attributed to protons of cholesterol in its free and esterified form, the headgroups and backbones of phospholipids and sphingolipids, the glycerol backbone and fatty acids—all of them being esterified (Figure 1). These constituents were quantified from well-resolved signals in the NMR fingerprint, as are listed in Supplementary Table S1 and constitute the targeted analysis. For the untargeted multivariate analysis, the buckets resulting from the division of the NMR spectra were initially explored by the unsupervised principal component analysis (PCA) in order to check the consistency and the quality of the data (data not shown).

### 2.3. T2DM vs. Control Model

**Figure 2:**
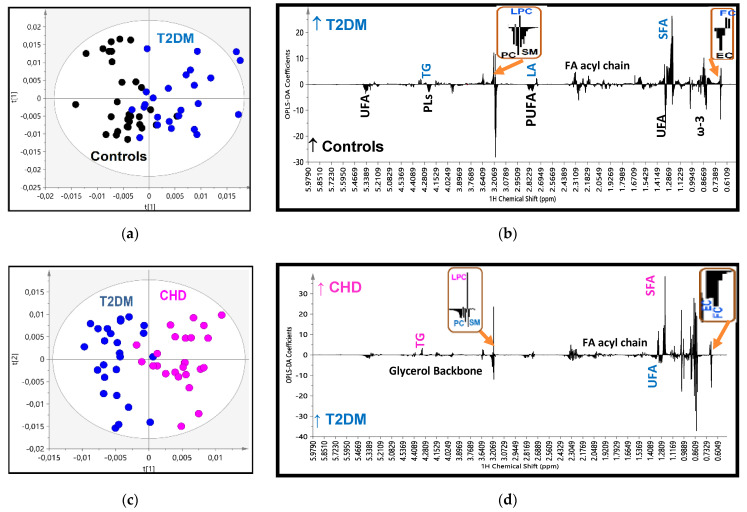

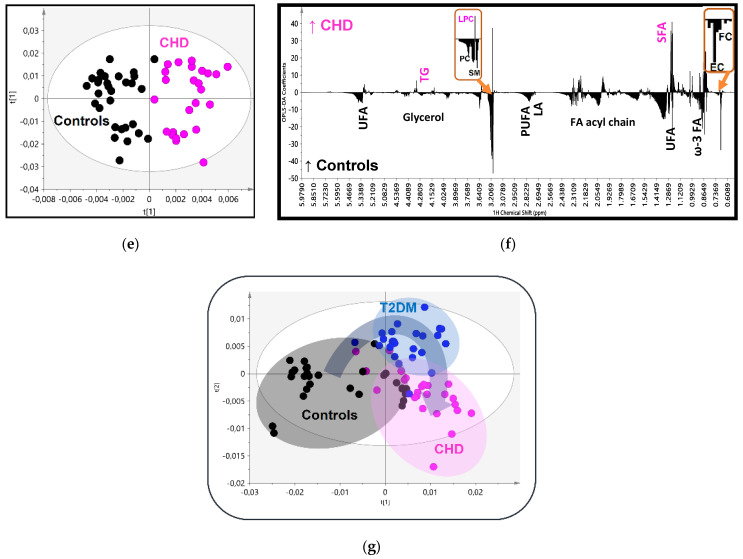
(**a**) OPLS-DA score plot of the HDL lipidomic data from 25 patients with Type 2 Diabetes (T2DM) (blue circles) and 30 controls (black circles); (**b**) the corresponding regression coefficient plot; (**c**) OPLS-DA score plot of the 25 patients with CHD (pink circles) and 30 controls (black circles); (**d**) the corresponding regression coefficient plot; (**e**) OPLS-DA score plot of the 25 patients with T2DM (blue circles) and 25 patients with Cononary Heart Disease (CHD) (pink circles); (**f**) the corresponding regression coefficient plot; and (**g**) OPLS-DA score plot of the three groups together.

In the OPLS-DA analysis, after reducing data dimensions to the principal components, the score plot for the T2DM vs. control model (Figure 2a) showed clustering into separate groups with good goodness of fit and predictive power (R^2^X = 0.629, R^2^Y = 0.538, Q^2^ = 0.251 and *p* < 0.05), and a minimal area of overlap. The analysis of the loading coefficient plot of the model, that means the relative significance of each constituent in the groups’ separation, shown in Figure 2b and Table 2, revealed that the determining factors were firstly, the lower levels of phosphatidylcholine (PC), sphingomyelin (SM) and cholesterol esters (CE) in HDL of diabetics, and secondly their higher levels of lysophosphatidylcholine (LysoPC) and those of free cholesterol (FC) and core triglycerides (TG) with a lower coefficient of significance. Furthermore, HDLs in T2DM patients were found to be enriched in saturated fatty acids (SFA) and depleted in polyunsaturated fatty acids (PUFA) (especially ω-3 fatty acids). Thereafter, we performed a quantitative targeted analysis of individual lipid constituents summarized in Table 3. The results in the targeted analysis are nearly totally consistent with those found in the untargeted multivariate analysis. Thus, compared to controls, T2DM patients displayed significantly lower concentrations of esterified (and subsequently total) cholesterol, PC and SM and significantly higher concentrations of lysoPC, FC and core TG. The higher ratios of FC/PC and FC/SM resulted subsequently in an increase of the Free cholesterol to Phopspholipids ratio (FC/PLs) ratio on the particles’ surfaces. In the targeted analysis we were able to quantify minor HDL constituents and fatty acids’ features (Table 3). T2DM patients had significantly lower plasmalogen phospholipid and phosphatidylethanolamine concentrations in their HDLs and higher levels of linoleic acid compared to controls, which possibly indicates low activities of D5D and D6D desaturases. However, a targeted lipidomic approach showed that T2DM patients presented with lower levels of linoleic acid compared to controls. The apparent inconsistency in the two different methods used is probably due to the fact that the bucket of LA does not represent the sum of its signal.

The analysis of score plot for the second model: the CHD vs. T2DM showed clustering into separate groups with good fit and predictive power (R^2^X = 0.524, R^2^Y = 0.744, Q^2^ = 0.408 and *p* < 0.001) and minimal overlap (Figure 2c). The determining factors in the separation are quoted in Figure 2d and Table 2. It is notable that in this model the determining constituents found previously in the T2DM vs. control model were further deteriorated towards the same direction as in CHD patients, e.g., PC, SM and CE were further reduced; lysoPC and core TG were further increased. FC was the only exception and was found lower in CHD compared to T2DM. The qualitative features of fatty acids in CHD were also further enriched in saturated FA and subsequently depleted in total and individual unsaturated fatty acids. The quantitative targeted analysis of individual lipid constituents (Table 3) confirmed the results derived from the loading coefficients. Thus, PC, SM, CE and unsaturated fatty acids are depleted in the HDLs of patients with CHD, whereas their contents in lysoPC and core TG are enhanced. Total cholesterol is reduced in CHD patients compared to T2DM mainly due to the reduction in FC content. The fluctuation in TG levels could possibly attributed to the large SD they present in T2DM and CHD patients despite the statistical significance.

Finally, the analysis of the CHD patients vs. control model resulted as expected with an optimum separation, high OPLS-DA parameters (R^2^X = 0.819, R^2^Y = 0.804, Q^2^ = 0.428 and *p* < 0.01) and high statistical significance in the HDLs composition, as it has been analyzed above (Figure 2e–f, Table 2 and Table 3).

This progressive alteration in the HDLs composition as previously analyzed from controls to T2DM and then to CHD is better depicted in the score plot model created for the three groups simultaneously (Figure 2g), suggesting a relatively high impact of these disorders on HDL composition.

The increasing interest in elucidating HDL functions and composition has greatly contributed to our understanding of HDL’s role in CVD and has strongly pointed out the need for the identification of new HDL metrics other than cholesterol that will be clinically relevant. New and sophisticated methodologies for assessing HDL functionality, composition and physiochemical properties have been proposed to aid in this effort. Most of these methodologies are research assays and not diagnostic tests routinely performed in a clinical setting. The lack of their standardization has led to conflicting results and discrepancies among HDL research studies. In addition, with the exception of the cholesterol efflux capacity assay, most of methods have not been tested in large numbers of subjects [8,9,18,19].

In this study, we applied the emerging methodology of NMR-based lipidomic analysis to explore the lipid compositions of HDLs in patients newly diagnosed with T2DM with normal conventional lipid parameters and compared them with the HDL lipid composition in patients with CVD.

High-field NMR has become a sophisticated and powerful analytical technology in areas of medical and pharmaceutical research to assess the molecular compositions of biosamples and in profiling complex diseases using multivariate metabolomics strategies [17]. The method provides a non-invasive “window” to biochemical processes within the body. Starting from the field of research, its applications are now expanding in clinical practice. It typically is conducted at high magnetic field strengths (≈11–14 T) for the simultaneous multicomponent analysis of complex bιοfluids, cell extracts and tissue samples, and at lower field strength along with whole-body magnetic resonance imaging (MRI), thereby obtaining, in addition to anatomical features, information on the metabolism of well-defined regions in the human body “in vivo” [20].

NMR is a promising new technique for expanding our understanding of the interrelationship between lipoprotein metabolism and CVD and has been successfully applied to quantify lipoprotein subfractions, providing both concentration and average size information, and receiving wide clinical acceptance [21,22]. The new and rapidly expanding benchtop low-field NMR instruments for chemical and biochemical analysis will likely contribute further to personalised medicine, clinical chemistry-based disease monitoring applications and the “point-of-care” diagnostic and prognostic screening of biofluids [23].

The main findings in the present study are the HDL particles’ core enrichment in TGs; the depletion of CE; the depletion of the main surface phospholipids, SM and PC; the increase in LysoPC content; and the partial replacement of unsaturated fatty acids with saturated ones. These alterations are gradually seen from controls to T2DM and then to CVD patients.

Elevated cholesteryl ester transfer protein (CETP), which mediates the hetero-exchange of TG and CE between VLDL and HDL, primarily accounts for this frequent abnormality in HDL. CETP activity is elevated in the dyslipidaemias of metabolic disease involving insulin resistance and moderate to marked hypertriglyceridaemia, and is intimately associated with premature atherosclerosis and high cardiovascular risk [24]. TG-enriched HDLs are less stable than normal particles and undergo more rapid degradation through TG hydrolysis by hepatic lipase [25]. The conformation of apoA1, the primary protein constituent of HDL, also appears to be very sensitive to the nature of HDL core neutral lipids [26]. The α-helix stability of apoAI is enhanced by CE but reduced by TG, whereby apoA1 dissociates from HDL and is cleared from the plasma [27]. Enrichment of HDL with TG has been shown to affect the process of reverse cholesterol transport (RCT) [28,29,30,31]. However, and beyond the RCT, these particles negatively influence other important metabolic pathways involved in heart disease, including a reduced capacity for lecithin; less cholesterol acyltransferase (LCAT) to convert free cholesterol to cholesterol esters; and a diminished capacity to deliver cholesterol esters to hepatic cells via SR-BI receptor for excretion in the bile [28,32]. The reduced ability of LCAT, as above mentioned, is also enhanced by the TG-induced, conformationally altered apoAI, which is the principal physiological co-factor for its action.

The phospholipid composition of HDLs is also a major determinant of cholesterol efflux. The surface rigidity of HDLs, partially regulated by the relative proportions of SM and FC in their lipid monolayer [33,34], influences the capacity of HDLs to serve as an acceptor of cholesterol [35]. Modifications in HDL phospholipids can affect both scavenger receptor class B member 1 (SR-BI) and ATP-binding cassette transporter (ABCA1)-mediated cholesterol efflux in a reciprocal manner [36]. Enrichment of HDLs with PC and SM enhances the bidirectional flux of cholesterol from SR-BI-expressing cells to HDL by two different mechanisms: PC increases the cholesterol efflux of cholesterol while SM decreases the influx [37]. Phospholipids also affect the ability of HDL to inhibit the cytokine-mediated increase in endothelial cell expression of adhesion molecules [38]. This process, known as inhibitory activity of HDL, may contribute to their antiatherogenic potential. PC was thought to be the main lipid component responsible for the inhibitory activity of HDLs. However, recent data showed particles containing SM were more efficient at inhibiting the release of TNF-α, IL-6 and IL-1β compared to those containing PC [38]. Finally, SM content of HDL is an important determinant of surface fluidity and RCT, and is negatively associated with future cardiovascular risk [39].

The HDL-lysoPC was higher in both our patient groups compared to controls, potentially reflecting enhanced hydrolysis of HDL lipids since this proinflammatory phospholipid subclass is generated primarily by the action of phospholipases on the plasma membrane and lipoprotein PC. Enrichment of HDL in lysoPC, which acts as a cell signaling molecule, may be relevant to the impaired biological activities of HDL in these patients [40]. In vitro data have confirmed the deleterious effects of lysoPC on HDL functionality, possibly via dissociation of apoA1 from HDL, consistent with detergent-like, protein-solubilizing properties of that lipid [41]. Rached et al. [42] have reported that HDL3b subpopulations in early-phase ST segment elevation myocardial infarction are enriched in lysoPC and phosphatidic acid, depleted in ApoA1 and enriched in serum amyloid A. These changes in the HDL lipidome together with changes in the HDL proteome contributed to functional deficiencies in cholesterol efflux and antioxidative activities of the circulating HDLs in these patients [42].

The fatty acid pattern of lipoproteins reflects dietary fatty acid intake but is also influenced by endogenous metabolic processes. Essential fatty acids such as omega-3 fatty acids are strongly affected by diet, whereas other fatty acids such as SFA can be synthesized de novo from acetyl coenzyme A. In the current study, SFA content was higher and UFA lower in T2DM, a pattern more intensively manifested in CVD patients. Data from experimental and clinical studies have shown that the fatty acid pattern strongly affects endothelial function. SFA induce proinflammatory responses [43], increase endothelial injury and impair endothelial repair capacity [44], whereas PUFA significantly improve endothelial function. In addition, the anti-inflammatory activity of HDLs, which includes the inhibition of the expression of adhesion molecules by cytokine-stimulated endothelial cells, is improved after the consumption of PUFA and reduced when the SFA content of the diet is increased [45]. The fatty acid profile can also be affected by endogenous desaturation catalyzed by desaturase enzymes such as Δ5-desaturase (D5D) and Δ6-desaturase (D6D), which have been found to predict the development of T2DM [46]. D5D enzyme catalyzes the desaturation of the C5–C6 bond in fatty acids, while the D6D catalyzed desaturation of the C6 to C7 bond, leading to the formation of ω-3 and ω-6 PUFA. The association of D5D and D6D activities with the risk of incident T2DM has been investigated in previous prospective studies, the majority of which found that low D5D activity enhanced T2DM risk, whereas the results about D6D activity in T2DM are conflicting [46,47]. In our study, T2DM had lower levels of the sum of eicosapentaenoic and arachidonic acid, and higher levels of linoleic acid in HDLs compared to controls, which possibly indicates low activities of D5D and D6D desaturases.

The key mechanisms that underlie the observed alterations in HDL lipid composition in T2DM patients or established CHD are not fully understood. Dysfunctional HDL may further promote vascular injury, thereby establishing a vicious cycle ultimately leading to plaque rapture. Indeed, in our study, numerically more patients in the CHD group were smokers, had a history of hypertension and had a family history of CHD. In addition, the inflammatory changes that take place during an acute coronary event may significantly affect the composition and properties of HDLs. Indeed, it has been proposed that ischemic events result in the remodeling of HDLs in a way that alters their antiatherogenic properties [48,49]. Therefore, the combination of lipid abnormalities and inflammatory milieu may establish a vicious circle that accelerates the atherosclerotic process of the vascular wall.

## 3. Materials and Methods

### 3.1. Subjects

Twenty-five patients with a confirmed diagnosis of myocardial infarction without persistent elevation of the ST segment NSTEMI (non-ST-elevation myocardial infarction); CHD group with angiographically demonstrated three-vessel disease) and 25 patients with T2DM diagnosed on admission and normal coronary arteries who were admitted to the Coronary Care Unit of the University Hospital of Ioannina participated in the study. The control group comprised 30 consecutive patients without T2DM who were admitted to the hospital because of episodes of atypical chest pain without any increase in biochemical markers, met the inclusion and exclusion criteria of the study (as defined below) and had angiographically normal coronary arteries. All patients underwent diagnostic coronary angiography within 7–9 days of the onset of the symptoms.

The diagnosis of NSTEMI was based on the criteria proposed by the European Society of Cardiology [50]. Patients who did not meet the criteria for NSTEMI after the initial evaluation were excluded from the study. The diagnosis of T2DM was based on fasting glucose and glycated hemoglobin (HbA_1c_) values measured on admission according to the American Diabetes Association criteria [51]. To avoid the confounding effects of glucose-lowering medications or extreme glycemic dysregulation on our results, we enrolled only patients with undiagnosed diabetes who had HbA1c above the threshold for T2DM diagnosis but below 8%. We included all patients with T2DM without CHD, irrespectively of their age, but in the analysis, we used only those that matched with CHD group for age and lipid parameters.

Patients with a history of CHD, chronic renal disease, hepatic function impairment, overt hyper-/hypothyroidism or rheumatic diseases; patients on lipid lowering drugs such as statins; patients on blood pressure lowering drugs that affect lipid metabolism (such as diuretics or beta blockers); patients on lipid lowering drugs; and patients with a history of diabetes mellitus were excluded from the study. Some of the individuals in the CHD group had metabolic syndrome and/or prediabetes, but none fulfilled the classical criteria for the diagnosis of overt T2DM (i.e., fasting glucose greater than 126 mg/dl and/or HbA1c greater than 6.4%).

For all participants, demographic characteristics, smoking habits, personal history regarding the presence of hypertension and family history of premature cardiovascular disease were recorded. All groups were matched according to age and serum lipid profile (total, LDL, non-HDL and HDL-cholesterol, triglyceride, apolipoprotein AI (ApoAI) and apolipoprotein B (ApoB) levels) to minimize the confounding effects of these parameters on the data analysis (Table 1).

Collection of samples from all participants was conducted in accordance with the guidelines of the Scientific Committee of the University Hospital of Ioannina. Written consent was obtained from each participant before any study procedure was performed. The study was approved by the Ethics committee of the University Hospital of Ioannina.

### 3.2. Sample Collection

Fasting venous blood samples were obtained in the morning before angiography for all study participants and in CHD patients within the first 12 h from the onset of symptoms to avoid lipoprotein changes. Serum was separated by centrifugation at 1500 *g* for 15 min for the determination of biochemical parameters and one 1.5 mL aliquot was stored at −80 °C until NMR analysis.

### 3.3. Biochemical Parameters

Total cholesterol (C) and triglycerides (TG) were determined enzymatically, and HDL-C by a direct assay on an Olympus AU5400 Clinical Chemistry analyzer (Beckman, Hamburg, Germany). LDL-C was calculated by the Friedewald formula, and non-HDL-C was calculated as the difference between total C and HDL-C. Serum apoAI and apoB were measured by immunonephelometry on a BN ProSpec System (Siemens, Marburg, Germany). HbA_1c_ was measured in ion exchange HPLC system (Variant II, Bio-Rad Laboratories, Hercules, CA, USA).

### 3.4. Isolation and lipid extraction of HDL lipoproteins

HDLs were separated from non-HDL particles by precipitation with dextran sulfate/MgCl_2_. The HDL lipid content was extracted according to the modified Bligh and Dyer method [52], as described previously chapter [16].

### 3.5. ^1^H NMR spectroscopy

The extracted HDL lipids were dissolved in 500 μL of deuterated methanol/chloroform (2:1, *v*/*v*). All the NMR spectra were recorded on a Bruker Avance DRX 600MHz NMR Spectrometer at 298 K. A “zgpr” Bruker pulse program was applied with the parameters as follows: 64 scans, a 90° pulse, a relaxation delay of 4 s and a 7184 Hz spectral width. The 128 free induction decays (FIDs) were multiplied by an exponential weighting function corresponding of 0.3 Hz line-broadening factor and Fourier transformed into 32 K data points. NMR spectra were phase and baseline corrected and referenced to the methanol signal (δ = 3.30 ppm) using TopSpin 2.1 software (Bruker Biospin Ltd, GmbH, Rheinstetten, Germany).

Quantification of the HDL lipids was based on the integration of selected signals in the proton NMR spectrum, corrected for the number of protons and then normalized with respect to the signal from the cholesterol C18 methyl group at 0.68 ppm. The lipid compositions of HDL lipoproteins were expressed as percentages of the total lipids of HDL particles. Spectral assignments and quantification of HDL lipid peaks were described previously [16].

### 3.6. Statistical analysis

All data are expressed as mean values ± SDs. Group comparisons were performed using one-way analysis of variance (ANOVA) followed by least significance differences (LSD) test for pairwise comparisons. A *p*-value < 0.05 was considered to indicate statistical significance.

The unsupervised (principal component analysis, PCA) and supervised (orthogonal projections to latent structures discriminant analysis, OPLS-DA) multivariate techniques were used to construct pattern recognition models to extract specific atherogenic lipidomic signatures of HDL in newly diagnosed T2DM patients.

NMR spectra were divided into 0.002 ppm width buckets over the chemical shift range (0.5; 6.0 ppm) using the AMIX 3.9 software (Bruker Biospin Corporation). All data were normalized to the total spectrum area and mean-centered prior to multivariate data analysis, carried out with the SIMCA-P+ 14 software (Umetrics, Umea, Sweden). Initial exploration of the lipidomic data was performed with principal component analysis (PCA) to identify possible groupings, trends and potential outliers (technical or biological) before supervised multivariate analysis using orthogonal projections to latent structures discriminant analysis (OPLS-DA). The OPLS-DA analysis eliminated the uncorrelated systematic variation and described the maximum separation based on class membership. The OPLS-DA score plot was used to show observations lying outside the 0.95 Hotteling’s T2 ellipse and to detect any grouping trend or separation, whereas the OPLS-DA loading coefficient plot was used to show the contributions of all NMR spectral regions or variables (corresponding to lipid constituents) to the grouping trend or separation seen in the OPLS-DA score plot. The performance of the OPLS-DA model was assessed by goodness-of-fit parameters R^2^ (R^2^X and R^2^Y) and Q^2^, related respectively to the explained and predicted variance calculated through 7-fold cross-validation. Cross-validated analysis of variance (CV-ANOVA) was also used to assess the significance of the OPLS-DA model. When the CV-ANOVA *p*-value was <0.5, the OPLS-DA model was considered reliable.

## 4. Conclusions

We conclude that patients with T2DM and normal serum lipid profiles, even at diagnosis, present significant alterations in the lipid composition of HDL, with atherogenic features that may be involved in the pathogenesis of atherosclerosis. These changes are qualitatively similar to those found, more profoundly, in normolipidemic patients with established CHD.

NMR-based lipidomics offer a novel holistic exploratory approach for identifying and quantifying lipid species in biological matrixes in physiological processes and disease states or in disease biomarker discovery.

## Figures and Tables

**Table 1:** Clinical and biochemical characteristics of the study population.

	Controls	Patients with T2DM	Patients with CHD
*n*	30	25	25
Age (years)	61.3 ± 7.7	63.3 ± 9.2	66.2 ± 10.8
Gender (M/F)	17/13	15/10	20/5
Hypertension (n)	6	11	15
Body Mass Index (Kg/m^2^)	26.5 ± 1.2	27.2 ± 2.1	26.2 ± 4.2
Current Smokers (*n*)	6	6	11
Metabolic syndrome (*n*)	7	23 ^§^	16 ^§ #^
Number of metabolic syndrome criteria (*n*)	2.1 ± 0.9	3.1 ± 0.5 ^†^	3 ± 1.1 ^†^
Family history of premature CVD (*n*)	2	1	7
Total cholesterol (mg/dL)	192.4 ± 41.6	215.0 ± 45.0	189.7 ± 34.3
Triglycerides (mg/dL)	114.7 ± 40.9	122.2 ± 43.5	137.8 ± 45.7
HDL-Cholesterol (mg/dL)	44.9 ± 9.3	48.4 ± 10.0	42.8 ± 6.6
LDL-Cholesterol (mg/dL)	124.5 ± 35.3	142.1 ± 40.0	119.3 ± 29.1
non-HDL-Cholesterol (mg/dL)	147.5 ± 38.9	166.6 ± 38.6	146.9 ± 32.3
Apolipoprotein AI (mg/dL)	131.1 ± 23.5	136.5 ± 21.0	121.5 ± 21.5
Apolipoprotein B (mg/dL)	91.0 ± 23.4	99.5 ± 20.9	91.4 ± 24.3
Fasting glucose (mg/dL)	93.9 ± 8.7	151.8 ± 25.4 *	99.4 ± 11.9
HbA_1c_ (%)	4.7 ± 0.6	7.3 ± 0.5 *	5.0 ± 0.4

Entries are mean ± SD, CHD = coronary heart disease; ^§^ *p* < 0.05 and ^#^ *p* < 0.05 vs. control and type-2 diabetes mellitus group, respectively (by Yates-corrected *x*^2^ test); ^†^ *p* < 0.01 vs. control group; * *p* < 0.001 vs. control and CHD groups.

**Table 2:** Untargeted analysis (relative significance in the groups’ separation) of HDL lipoproteins of the studied groups.

	T2DM vs. Controls		CHD vs. T2DM		CHD vs. Controls	
**Lipid Constituent**	change	Coef	change	Coef	change	Coef
**Structural Components**						
EC	↓	13.52	↓	12.56	↓	33.57
FC	↑	6.35	↓	15.88	↓	10.01
TG	↑	2.11	↑	3.36	↑	6.81
PLs	↓	3.03	↓	0.40	↓	1.91
PC	↓	28.16	↓	8.52	↓	36.55
SM	↓	20.40	↓	11.94	↓	47.09
LysoPC	↑	11.66	↑	23.58	↑	37.41
PE	↑	0.80	↓	0.40	↓	0.40
Plasmalogen	↓	0.70	↑	0.10	↓	0.20
**Qualitative Features of Fatty Acids**						
SFA	↑	26.40	↑	38.54	↑	41.04
UFA	↓	17.41	↓	3.90	↓	15.54
PUFA	↓	2.66	↓	1.43	↓	5.02
ω-3 FA	↓	2.88	↓	2.49	↓	4.18
LA	↑	2.38	↑	1.85	↓	4.40
EPA + AA	↓	2.02	↓	0.16	↓	2.13
DHA	↓	0.66	↑	1.50	↑	2.15

**Table 3:** Targeted analysis (lipid quantification) of HDL lipoproteins of the studied groups.

	Controls	T2DM	CHD
**% Lipid Constituent ^a^**	mean ± SD		
**Structural Components**			
% TC	41.05 ± 2.38	38.77 ± 3.32 **	36.35 ± 2.45 **^++^
% EC	31.99 ± 2.11	29.16 ± 3.39 **	27.74 ± 3.11 **
% FC	9.06 ± 1.11	9.61 ± 1.56	8.61 ± 1.61 *^+^*
% TG	9.00 ± 1.27	13.34 ± 5.05 **	15.16 ± 5.63 **
% PLs	49.94 ± 2.44	47.89 ± 3.51 *	48.49 ± 4.30
% PC	32.55 ± 2.45	29.52 ± 4.49 **	28.84 ± 5.10 **^+^
% SM	7.71 ± 1.10	6.73 ± 1.31 **	6.69 ± 1.15 **
% LysoPC	1.94 ± 0.53	3.75 ± 1.56 **	4.13 ± 1.50 **
% PE	1.44 ± 0.59	0.95 ± 0.30 **	0.76 ± 0.17 **
% PI+PS	4.68 ± 3.52	5.72 ± 3.26	4.79 ± 2.45
% Plasmalogens	1.62 ± 0.34	1.22 ± 0.36 **	1.28 ± 0.47 **
% Core	40.99 ± 2.40	42.50 ± 3.56	42.90 ± 4.05
% Surface	59.01 ± 2.40	57.50 ± 3.56	57.10 ± 4.05
Ratio PC/SM	4.31 ± 0.77	4.54 ± 1.04	4.71 ± 1.02
Ratio EC/FC	3.58 ± 0.47	3.12 ± 0.62 *	3.39 ± 1.00
Ratio FC/PLs	0.18 ± 0.03	0.20 ± 0.04 *	0.18 ± 0.04
Ratio FC/PC	0.28 ± 0.04	0.33 ± 0.07 **	0.29 ± 0.09
Ratio FC/SM	1.19 ± 0.19	1.48 ± 0.42 **	1.34 ± 0.42
**Qualitative Features of Fatty Acids**			
% SFA	39.83 ± 4.10	41.39 ± 4.96 *	49.14 ± 7.26 **^++^
% UFA	60.17 ± 4.10	59.61 ± 4.96	50.86 ± 7.26 **^++^
% MUFA	14.85 ± 6.35	13.19 ± 6.63	9.39 ± 5.02 **^+^
% PUFA	45.32 ± 6.32	43.42 ± 3.84 *	41.47 ± 7.39 *^+^
% LA	20.83 ± 3.05	19.76 ± 3.43 *	17.54 ± 3.31 **^++^
% EPA + AA	12.47 ± 2.33	12.28 ± 4.16	12.38 ± 2.13
% DHA	3.90 ± 0.80	3.95 ± 0.80	3.83 ± 0.66

^a^ Expressed as moles/100 moles of total lipid content. * *p* < 0.05 and ** *p* < 0.01 compared to controls; ^+^ *p* < 0.05 and ^++^ *p* < 0.01 compared to T2DM.

## Supplementary Materials

The following are available online at https://www.mdpi.com/1422-0067/21/22/8835/s1.

## Abbreviations

AA	Arachidonic Acid
DAGPLs	Diacyl Glycerophospholipids
DHA	Docosahexaenoic Acid
EC	Esterified Cholesterol
EPA	Eicosapentaenoic Acid
FA	Fatty acids
FC	Free Cholesterol
GPLs	Glycerophospholipids
LA	Linoleic Acid
LysoPC	Lysophsphatidylcholine
MUFA	Monounsaturated Fatty Acids
PC	Phosphatidylcholine
PE	Phosphatidylethanolamine
PI	Phosphatidylinositol
PLA	Plasmalogens
Pls	Phospholipids
PS	Phosphatidylserine
PUFA	Polyunsaturated Fatty Acids
SFA	Saturated Fatty Acids
SLs	Sphingolipids
SM	Sphingomyelin
TC	Total Cholesterol
TG	Triglycerides
UFA	Unsaturated Fatty Acids
ω-3 FA	Total Omega-3 Fatty Acids

## References

[1] Haffner S.M., Lehto S., Ronnemaa T., Pyorala K., Laakso M. (1998). Mortality from coronary heart disease in subjects with type 2 diabetes and in nondiabetic subjects with and without prior myocardial infarction. N. Engl. J. Med..

[2] Bulugahapitiya U., Siyambalapitiya S., Sithole J., Idris I. (2009). Is diabetes a coronary risk equivalent? Systematic review and meta-analysis. Diabetes Med..

[3] Stamler J., Vaccaro O., Neaton J.D., Wentworth D. (1993). Diabetes, other risk factors, and 12-yr cardiovascular mortality for men screened in the Multiple Risk Factor Intervention Trial. Diabetes Care.

[4] Turner R.C., Millns H., Neil H.A.W., Stratton I.M., Manley S.E., Matthews D.R., Holman R.R. (1998). Risk factors for coronary artery disease in non-insulin dependent diabetes mellitus: United Kingdom Prospective Diabetes Study (UKPDS: 23). BMJ.

[5] Vollenweider P., von Eckardstein A., Widmann C. (2015). HDLs, diabetes, and metabolic syndrome. Handbook of Experimental Pharmacology.

[6] Steiner G. (1994). The dyslipoproteinemias of diabetes. Atherosclerosis.

[7] Karathanasis S.K., Freeman L.A., Gordon S.M., Remaley A.T. (2017). The Changing Face of HDL and the Best Way to Measure It. Clin. Chem..

[8] Niisuke K., Horvath K.V., Asztalos B.F. (2018). Where next with HDL assays?. Curr. Opin. Lipidol..

[9] Cardner M., Yalcinkaya M., Goetze S., Luca E., Balaz M., Hunjadi M., Hartung J., Shemet A., Kränkel N., Radosavljevic S. (2020). Structure-function relationships of HDL in diabetes and coronary heart disease. JCI Insight.

[10] von Eckardstein A., Rohrer L. (2016). HDLs in crises. Curr. Opin. Lipidol..

[11] Hui N., Barter P.J., Ong K.L., Rye K.A. (2019). Altered HDL metabolism in metabolic disorders: Insights into the therapeutic potential of HDL. Clin. Sci..

[12] Kontush A., Lhomme M., Chapman M.J. (2013). Unraveling the complexities of the HDL lipidome. J. Lipid Res..

[13] O’Donnell V.B., Ekroos K., Liebisch G., Wakelam M. (2020). Lipidomics: Current state of the art in a fast moving field. Wiley Interdiscip. Rev. Syst. Biol. Med..

[14] Ding M., Rexrode K.M. (2020). A Review of Lipidomics of Cardiovascular Disease Highlights the Importance of Isolating Lipoproteins. Metabolites.

[15] Li J., Vosegaard T., Guo Z. (2017). Applications of nuclear magnetic resonance in lipid analyses: An emerging powerful tool for lipidomics studies. Prog. Lipid Res..

[16] Kostara C.E., Bairaktari E.T., Lutz N., Sweedler J., Wevers R.A. (2012). Lipid profiling in health and disease. Methodologies for Metabolomics: Experimental Strategies and Techniques.

[17] Markley J.L., Bruschweiler R., Edison A.S., Eghbalnia H.R., Powers R., Raftery D., Wishart D.S. (2017). The future of NMR-based metabolomics. Curr. Opin. Biotechnol..

[18] Heinecke J.W., Bornfeldt K.E. (2017). A Long Road Ahead for Discovering New HDL Metrics That Reflect Cardiovascular Disease Risk. J. Am. Coll. Cardiol..

[19] Asztalos B.F., Niisuke K., Horvath K.V. (2019). High-density lipoprotein: Our elusive friend. Curr. Opin. Lipidol..

[20] Tognarelli J.M., Dawood M., Shariff M.I., Grover V.P., Crossey M.M., Cox I.J., Taylor-Robinson S.D., McPhail M.J. (2015). Magnetic Resonance Spectroscopy: Principles and Techniques: Lessons for Clinicians. J. Clin. Exp. Hepatol..

[21] Otvos J.D., Collins D., Freedman D.S., Shalaurova I., Schaefer E.J., McNamara J.R., Bloomfield H.E., Robins S.J. (2006). Low-density lipoprotein and high-density lipoprotein particle subclasses predict coronary events and are favorably changed by gemfibrozil therapy in the Veterans Affairs High-Density Lipoprotein Intervention Trial. Circulation.

[22] Ala-Korpela M., Lankinen N., Salminen A., Suna T., Soininen P., Laatikainen R., Ingman P., Jauhiainen M., Taskinen M.R., Héberger K. (2007). The inherent accuracy of 1H NMR spectroscopy to quantify plasma lipoproteins is subclass dependent. Atherosclerosis.

[23] Grootveld M., Percival B., Gibson M., Osman Y., Edgar M., Molinari M., Mather M.L., Casanova F., Wilson P.B. (2019). Progress in low-field benchtop NMR spectroscopy in chemical and biochemical analysis. Anal. Chim. Acta.

[24] Chapman M.J., Le Goff W., Guerin M., Kontush A. (2010). Cholesteryl ester transfer protein: At the heart of the action of lipid-modulating therapy with statins, fibrates, niacin, and cholesteryl ester transfer protein inhibitors. Eur. Heart J..

[25] Lamarche B., Rashid S., Lewis G.F. (1999). HDL metabolism in hypertriglyceridemic states: An overview. Clin. Chim. Acta.

[26] Sparks D.L., Davidson W.S., Lund-Katz S., Phillips M.C. (1995). Effects of the neutral lipid content of high density lipoprotein on apolipoprotein A-I structure and particle stability. J. Biol. Chem..

[27] Curtiss L.K., Bonnet D.J., Rye K.A. (2000). The conformation of apolipoprotein A-I in high-density lipoproteins is influenced by core lipid composition and particle size: A surface plasmon resonance study. Biochemistry.

[28] Skeggs J.W., Morton R.E. (2002). LDL and HDL enriched in triglyceride promote abnormal cholesterol transport. J. Lipid Res..

[29] Fournier N., Francone O., Rothblat G., Goudouneche D., Cambillau M., Kellner-Weibel G., Robinet P., Royer L., Moatti N., Simon A. (2003). Enhanced efflux of cholesterol from ABCA1-expressing macrophages to serum from type IV hypertriglyceridemic subjects. Atherosclerosis.

[30] Yassine H.N., Belopolskaya A., Schall C., Stump C.S., Lau S.S., Reaven P.D. (2014). Enhanced cholesterol efflux to HDL through the ABCA1 transporter in hypertriglyceridemia of type 2 diabetes. Metabolism.

[31] Asztalos B.F., Horvath K.V., Mehan M., Yokota Y., Schaefer E.J. (2017). Influence of HDL particles on cell-cholesterol efflux under various pathological conditions. J. Lipid Res..

[32] Greene D.J., Skeggs J.W., Morton R.E. (2001). Elevated triglyceride content diminishes the capacity of high density lipoprotein to deliver cholesteryl esters via the scavenger receptor class B type I (SR-BI). J. Biol. Chem..

[33] Kontush A., Therond P., Zerrad A., Couturier M., Négre-Salvayre A., de Souza J.A., Chantepie S., Chapman M.J. (2007). Preferential sphingosine-1-phosphate enrichment and sphingomyelin depletion are key features of small dense HDL3 particles: Relevance to antiapoptotic and antioxidative activities. Arterioscler. Thromb. Vasc. Biol..

[34] Zerrad-Saadi A., Therond P., Chantepie S., Couturier M., Rye K.A., Chapman M.J., Kontush A. (2009). HDL3-mediated inactivation of LDL-associated phospholipid hydroperoxides is determined by the redox status of apolipoprotein A-I and HDL particle surface lipid rigidity: Relevance to inflammation and atherogenesis. Arterioscler. Thromb. Vasc. Biol..

[35] Davidson W.S., Gillotte K.L., Lund-Katz S., Johnson W.J., Rothblat G.H., Phillips M.C. (1995). The effect of high density lipoprotein phospholipid acyl chain composition on the efflux of cellular free cholesterol. J. Biol. Chem..

[36] Yancey P.G., Kawashiri M.A., Moore R., Glick J.M., Williams D.L., Connelly M.A., Rader D.J., Rothblat G.H. (2004). In vivo modulation of HDL phospholipid has opposing effects on SR-BI- and ABCA1-mediated cholesterol efflux. J. Lipid Res..

[37] Yancey P.G., de la Llera-Moya M., Swarnakar S., Monzo P., Klein S.M., Connelly M.A., Johnson W.J., Williams D.L., Rothblat G.H. (2000). High density lipoprotein phospholipid composition is a major determinant of the bi-directional flux and net movement of cellular free cholesterol mediated by scavenger receptor BI. J. Biol. Chem..

[38] Baker P.W., Rye K.A., Gamble J.R., Vadas M.A., Barter P.J. (2000). Phospholipid composition of reconstituted high density lipoproteins influences their ability to inhibit endothelial cell adhesion molecule expression. J. Lipid Res..

[39] Martinez-Beamonte R., Lou-Bonafonte J.M., Martinez-Gracia M.V., Osada J. (2013). Sphingomyelin in high-density lipoproteins: Structural role and biological function. Int. J. Mol. Sci..

[40] Schmitz G., Ruebsaamen K. (2010). Metabolism and atherogenic disease association of lysophosphatidylcholine. Atherosclerosis.

[41] Schwendeman A., Sviridov D.O., Yuan W., Guo Y., Morin E.E., Yuan Y., Stonik J., Freeman L., Ossoli A., Thacker S. (2015). The effect of phospholipid composition of reconstituted HDL on its cholesterol efflux and anti-inflammatory properties. J. Lipid Res..

[42] Rached F., Lhomme M., Camont L., Gomes F., Dauteuille C., Robillard P., Santos R.D., Lesnik P., Serrano C.V., Chapman M.J. (2015). Defective functionality of small, dense HDL3 subpopulations in ST segment elevation myocardial infarction: Relevance of enrichment in lysophosphatidylcholine, phosphatidic acid and serum amyloid A. Biochim. Biophys. Acta.

[43] Harvey K.A., Walker C.L., Pavlina T.M., Xu Z., Zaloga G.P., Siddiqui R.A. (2010). Long-chain saturated fatty acids induce pro-inflammatory responses and impact endothelial cell growth. Clin. Nutr..

[44] Marin C., Ramirez R., Delgado-Lista J., Yubero-Serrano E.M., Perez-Martinez P., Carracedo J., Garcia-Rios A., Rodriguez F., Gutierrez-Mariscal F.M., Gomez P. (2011). Mediterranean diet reduces endothelial damage and improves the regenerative capacity of endothelium. Am. J. Clin. Nutr..

[45] Nicholls S.J., Lundman P., Harmer J.A., Cutri B., Griffiths K.A., Rye K.A., Barter P.J., Celermajer D.S. (2006). Consumption of saturated fat impairs the anti-inflammatory properties of high-density lipoproteins and endothelial function. J. Am. Coll. Cardiol..

[46] Das U.N. (2010). A defect in Delta6 and Delta5 desaturases may be a factor in the initiation and progression of insulin resistance, the metabolic syndrome and ischemic heart disease in South Asians. Lipids Health Dis..

[47] Kroger J., Schulze M.B. (2012). Recent insights into the relation of Delta5 desaturase and Delta6 desaturase activity to the development of type 2 diabetes. Curr. Opin. Lipidol..

[48] Dullaart R.P., Annema W., Tio R.A., Tietge U.J. (2014). The HDL anti-inflammatory function is impaired in myocardial infarction and may predict new cardiac events independent of HDL cholesterol. Clin. Chim. Acta.

[49] Garcia C., Montee N., Faccini J., Series J., Meilhac O., Cantero A.V., Le Faouder P., Elbaz M., Payrastre B., Vindis C. (2018). Acute coronary syndrome remodels the antiplatelet aggregation properties of HDL particle subclasses. J. Thromb. Haemost..

[50] Roffi M., Patrono C., Collet J.P., Mueller C., Valgimigli M., Andreotti F., Bax J.J., Borger M.A., Brotons C., Chew D.P. (2016). 2015 ESC Guidelines for the management of acute coronary syndromes in patients presenting without persistent ST-segment elevation: Task Force for the Management of Acute Coronary Syndromes in Patients Presenting without Persistent ST-Segment Elevation of the European Society of Cardiology (ESC). Eur. Heart J..

[51] American Diabetes Association (2018). 2. Classification and Diagnosis of Diabetes: Standards of Medical Care in Diabetes-2018. Diabetes Care.

[52] Bligh E.G., Dyer W.J. (1959). A rapid method of total lipid extraction and purification. Can. J. Biochem. Physiol..

