# Supplementary material for: Early Signs of Atherogenic Features in the HDL Lipidomes of Normolipidemic Patients Newly Diagnosed with Type 2 Diabetes
Source: Int J Mol Sci. 2020 Nov 22;21(22):8835. doi: 10.3390/ijms21228835 (PMC7700318; doi:10.3390/ijms21228835)
Supplement: Supplementary file 1 [file ijms-21-08835-s001.pdf]

Table S1: Protons and Chemical Shift (in ppm) for the lipid constituents and headgroups identified in HDL lipid extract by NMR and selected signals for Lipid Quantification

**Lipid constituents and headgroups**	**^{1}H NMR signal assignment**	**Chemical Shift (ppm)**	**Quantification of lipids from selected well-resolved NMR signals**
**Cholesterol molecule**	$C_{18}\mathbf{H}_3$	0.68	**Total Cholesterol, FC, CE**
	$C_{26}\mathbf{H}_3$, $C_{27}\mathbf{H}_3$, $C_{21}\mathbf{H}$	0.87	
	$C_{19}\mathbf{H}_3$	1.00	
	$C_3\mathbf{H}$	3.40	
	$C_6\mathbf{H}$	5.36	
**Glycerol backbone**	$C_1\mathbf{H}^u$ and $C_3\mathbf{H}^u$ of glycerol backbone of TG and DAGPLs	4.16	
	$C_1\mathbf{H}^d$ and $C_3\mathbf{H}^d$ of glycerol backbone of TG	4.32	**TG**
	$C_1\mathbf{H}^d$ and $C_3\mathbf{H}^d$ of glycerol backbone of DAGPLs	4.40	
	$C_2\mathbf{H}$ of glycerol backbone in ether glycerophospholipids	5.15	**Ether GPLs**
	$C_2\mathbf{H}$ of glycerol backbone in total DAGPLs	5.18	**Total Diacyl glycerophospholipids (DAGPLs)**
	$C_2\mathbf{H}$ of glycerol backbone in TG	5.22	
**Sphingosine moiety**	$-CH_2-CH=C\boldsymbol{H}CHOH$	5.40	
	$-CH_2-C\boldsymbol{H}=CHCHOH$	5.70	**Total SLs**
**Head-group and substituent**	$-CH_2-CH_2-N^+(C\mathbf{H}_3)_3$ (choline)	3.20	**Total choline-containing PLs (PC, SM, LPC)**
	$-CH_2-C\mathbf{H}_2-N^+(CH_3)_3$	3.59	
	$-C\mathbf{H}_2-CH_2-N^+(CH_3)_3$	4.24	
	$-CH_2-C\mathbf{H}_2-NH_3^+$ (ethanolamine)	3.10	**PE**
	$-OC\mathbf{H}=CHCH_2$	5.90	**PLA (ether GPLs)**
**Fatty acid chains**	ω-$C\mathbf{H}_3$ (methyl) in fatty acyl chains	0.88	
	ω-$C\mathbf{H}_3$ (methyl) of total omega-3 FA	0.95	
	$-(C\mathbf{H}_2)_n-$ (methylene) in fatty acyl chains	1.30	
	$-CO-CH_2-C\mathbf{H}_2-$ (β-methylene) in the fatty acyl chains	1.59	
	β-$C\mathbf{H}_2$ (β-methylene) of the sum of AA+EPA	1.67	**AA (20:4 ω-6) + EPA (20:5 ω-3)**
	$-C\mathbf{H}_2-CH=$ (allylic) in fatty acyl chains	2.04	**UFA**
	$-CO-C\mathbf{H}_2$ (α-methylene) in the fatty acyl chains	2.30	**Total FA**
	α and β $C\mathbf{H}_2$ (methylene) of DHA	2.38	**DHA (22:6 ω-3)**
	$-CH=CH-C\mathbf{H}_2-CH=CH-$ of linoleic acid	2.75	**LA (18:2 ω-6)**
	$-(CH=CH-C\mathbf{H}_2-CH=CH)n$, $n > 1$ in the fatty acyl chains	2.80	**PUFA**
	$-C\mathbf{H}=C\mathbf{H}-$ in the fatty acyl chains	5.36	

Key AA, arachidonic acid; CE, cholesteryl ester; DAGPLs, Diacyl glycerophospholipids; DHA, docosahexaenoic acid; EPA, eicosapentaenoic acid; FA, fatty acids; FC, free cholesterol; GPLs, glycerophospholipids; LA, Linoleic acid; LPC, lysophosphatidylcholine; PC, phosphatidylcholine; PE, phosphatidylethanolamine; PLA, plasmalogens; PUFA, polyunsaturated fatty acids; SLs, Sphingolipids; SM, sphingomyelin; TG, triglycerides; UFA, Unsaturated fatty acids.
